# Tailoring Semantic Interventions for Older Adults: Task-Focused and Person-Centered Approaches

**DOI:** 10.3390/brainsci14090907

**Published:** 2024-09-07

**Authors:** Vasiliki Folia, Susana Silva

**Affiliations:** 1 Lab of Cognitive Neuroscience, School of Psychology, Aristotle University of Thessaloniki, 541 24 Thessaloniki, Greece; 2 Center for Psychology at University of Porto, Faculty of Psychology and Educational Sciences, University of Porto, 4200-135 Porto, Portugal; susanamsilva@fpce.up.pt

**Keywords:** linguistic–semantic interventions, cognitive intervention programs, person-centered approach, task-focused approach, older adults

## Abstract

In this narrative review, we explore the latest evidence on semantic interventions for older adults, including both prevention and rehabilitation/remediation efforts, discussing them particularly in the context of dementia. Cognitive interventions vary in their level of structure, encompassing standardized (task-focused tasks) and unstandardized tasks (person-centered tasks). These interventions also differ in their target: rehabilitation or prevention. Addressing semantic knowledge/semantic memory/semantics is important, primarily because its efficiency impacts other cognitive domains. Semantic tasks are commonly included in preventive and rehabilitation programs, typically as standardized tasks with pre-defined semantic referents. On the other hand, person-centered approaches introduce personally relevant semantics, allowing patients to share thoughts and experiences with expressive language. Although these approaches offer benefits beyond cognitive improvement, their lack of structure may pose challenges. Our question club (CQ) program blends structured activities with personally relevant semantics, aiming to harness the advantages of both methods. Additionally, in this narrative review, we discuss future challenges and directions in the field of semantic interventions.

## 1. Cognitive Interventions in Older Adults

The literature on cognitive programs includes various terms for different subtypes of cognitive interventions for older adults, such as cognitive stimulation, activation, training, rehabilitation, remediation, and enrichment, among others [1,2,3,4,5]. These programs are specifically designed to directly improve and maintain the cognitive function. The distinctions between these terms are not always clear, but some basic differences can be identified. One differentiator is the level of standardization in the activities: some approaches adhere to a script-based, controlled procedure (task-focused interventions), while others value the input provided by the participants (person-centered interventions), as seen, for example, in group discussions or reminiscence therapy [6,7]. Another differentiator is the target population: rehabilitation and remediation are primarily focused on brain-injured patients, while other approaches are more preventive and aim to support cognitive health in a broader population.

In this narrative review, we focus on a specific cognitive domain that is important to address as a goal—linguistic semantics—and on how it has been approached by task-focused versus person-centered interventions. While semantic therapy is a well-known cognitive rehabilitation activity for certain pathologies, such as aphasia [8,9,10], we know very little about the effectiveness of such programs in dementia prevention and rehabilitation/remediation. 

Therefore, our emphasis will be on semantic intervention programs for older adults, including both prevention and rehabilitation/remediation efforts within the scope of dementia. We aim to provide a comprehensive examination and discuss the current state of evidence on semantic cognitive interventions (both prevention and rehabilitation programs) targeted at older adults. First, we will explore the importance of semantics in cognitive interventions, emphasizing how semantic memory and knowledge are crucial for maintaining overall cognitive health. Next, we will delve into the outcomes of task-focused linguistic semantic training, highlighting its impact on various cognitive domains. Furthermore, the review will discuss the benefits of expressive language and person-centered semantic interventions. The review will not only synthesize the existing strengths of both task-focused and person-centered approaches but also propose new ideas, such as the novel question club (CQ) program, which integrates structured expressive language activities with person-centered semantics, aiming to combine the strengths of both approaches.

At the conclusion of this narrative review, we will consider future directions for research and practice in semantic interventions, addressing potential challenges and opportunities for enhancing cognitive health in older adults.

## 2. Methodology

### 2.1. Sources of Information

This narrative review synthesizes information mainly from a broad range of peer-reviewed articles and books. The databases used for this review were PubMed and SCOPUS. Additional sources were identified through manual searches of reference lists from key articles and related reviews.

### 2.2. Search Strategy and Keywords

The search strategy involved using specific keywords. The following keywords alone or in combination were used to search the databases: “linguistic semantic interventions”, “cognitive intervention programs”, “person-centered approach”, “task-focused approach”, “older adults”, “dementia”, “Mild Cognitive Impairment “, “Alzheimer’s Disease “, “cognitive stimulation”, “cognitive training”, “cognitive rehabilitation”. These terms were chosen to capture a wide array of studies focusing on both structured (task-focused) and unstructured (person-centered) cognitive interventions that target older adults, with particular focus in dementia.

### 2.3. Selection and Integration of the Sources

The inclusion criteria for selecting the sources were the following: (1) studies focusing on semantic interventions for older adults; (2) research on cognitive programs that explicitly target linguistic semantics; (3) articles discussing task-focused and person-centered approaches; (4) reviews or empirical studies published in English. Articles were excluded if they did not directly address semantic interventions, were not relevant to the older adult population, or were not relevant to dementia.

## 3. The Role of Semantics in Cognitive Interventions

The cognitive domains most frequently targeted in cognitive interventions for dementia are memory, attention, and executive functions [3,11,12,13]. However, language training, whether in preventive or in rehabilitation programs, is largely absent from metanalyses and systematic reviews examining the efficacy of cognitive prevention and rehabilitation training in healthy older individuals and dementia patients [14,15,16,17,18,19,20,21]. Among the goals related to long-term memory, preserving the subsystem of semantic memory is essential for maintaining an individual’s connection to the physical and social world [22,23]. Semantic memory, also referred to as conceptual or semantic knowledge, involves the general knowledge of objects, word meanings, facts, and people, collectively known as semantic representations. Unlike episodic memory, semantic memory is not tied to specific times and places [24]. An efficient semantic memory system must not only allow for agile access to stored information (retrieval), but also optimize forms of storage methods, which can, in turn, facilitate information retrieval. Semantic memory also plays a crucial role in daily life activities, such as managing finances. For example, in patients with mild cognitive impairment (MCI), these abilities have been shown to be impaired [25]. Retrieval abilities are demonstrated in tasks such as naming objects, defining words, or matching pictures with words. Category fluency, or the ability to name multiple elements within a given category (e.g., animals), is an example of a task related to storage [26]. Although it is possible to conceive of semantic memory tasks that do not involve language—for instance, matching one picture with another based on semantic similarity—most tasks do involve language. Therefore, most semantic memory training is also linguistic–semantic, or, as it has become popularly known, lexical–semantic [27].

Severe semantic memory deficits are not a common consequence of healthy ageing [28,29,30], although they are characteristic of conditions such as mild cognitive impairment (MCI) [31,32], Alzheimer’s disease (AD) [33], and semantic dementia (SD), which is a form of aphasia [34]. Interventions based on linguistic–semantic tasks have shown various positive effects in counteracting the age-related cognitive decline [35]. On one hand, the use of linguistic–semantic strategies—such as chunking or the hierarchical organization of information—is known to enhance the learning and retrieval of new information in healthy older adults and MCI patients [36,37,38,39]. On the other hand, various studies have shown that the cognitive benefits of linguistic–semantic training extend well beyond semantic memory, as we will show in the following section.

## 4. Task-Focused Approaches: Results from Linguistic–Semantic Training

Semantic tasks are an integral part of cognitive linguistic therapies, which originate from the cognitive linguistic approach focused on addressing language deficits [40]. As such, these tasks include activities that target general knowledge, the extraction of meaning from oral or written language (comprehension), and the storage of meaning-related information through processes such as categorization or chunking.

For example, the linguistic–semantic intervention program BOX [41], originally developed to rehabilitate aphasic patients, has also demonstrated positive outcomes in cognitive measures of episodic memory in early Alzheimer’s disease (AD) patients, as well as improvements in other cognitive areas. These cognitive outcomes are measured using tools like the Mini-Mental State Examination (MMSE), the Boston Naming Test (BNT), the Verbal Naming Test (VNT), the Brief Story Recall, the Stroop test and the Rey Auditory Verbal Learning Test (RAVL) delayed recall mean scores [42,43]. BOX incorporates various semantic decision tasks designed to enhance semantic processing. Each task features different types of exercises where patients confirm or deny the semantic relationship between content words presented either in written or in auditory form, either separately or within sentences or texts. The program emphasizes the interpretation of written words, sentences, and texts, with auditory presentations by a speech and language therapist when necessary. The design of this intervention program considers factors such as word choice (i.e., imageability, frequency, word length, and abstractness are considered), number of distractors (i.e., the difficulty level generally increases by adding more distractors), semantic relatedness (easier levels contain mostly unrelated distractors, while the most difficult levels contain only related distractors), and ambiguity (the difficult levels incorporate ambiguous words, requiring patients to interpret their meanings simultaneously) to create different levels of difficulty [41].

In addition to programs like BOX [41], where all activities relate to semantic processing and serve as examples of cognitive linguistic therapy, several other intervention programs integrate semantic processing tasks with activities targeting other cognitive functions, such as executive functions or visuospatial memory, evaluated through relevant cognitive measures.

Prevention intervention programs including tasks related to general knowledge, as well as oral and written language comprehension, have been shown to enhance cognitive functioning in healthy older adults, particularly in cognitive outcomes measuring working memory, processing speed, and learning potential [11,44].

Rehabilitation intervention programs using semantic tasks have been studied in dementia. Savage et al. [34] investigated whether semantic dementia patients can relearn the names of objects and transfer these relearned words to contexts other than picture naming through a rehabilitation task. This task involved repetitive training that paired the target-item photos with labels, both in written form and through audio recordings of the spoken word. The generalization of these relearned words beyond the specific task was assessed using both expressive and receptive language tasks. The results indicated evidence of generalization effects, particularly in patients with milder semantic impairments.

In general, rehabilitation programs incorporating verbal-learning exercises, picture recognition, word recall, reading aloud, sentence completion, and proverb explanation tasks have shown to enhance cognitive functioning in AD patients [45,46,47], particularly in areas such as attention, working memory, language comprehension, and executive functions. The cognitive rehabilitation program Brainer (https://www.brainer.it/) [48] was evaluated in two randomized controlled trials (RCTs) involving early-stage AD patients [45,46]. The studies found that the intervention had a positive impact on working memory, language comprehension, and executive functions, with these effects persisting for six months after the intervention but diminishing after twelve months. The study of Trebastoni et al. [47] evaluated the effectiveness of a cognitive training program in AD patients, which involved twice-weekly group cognitive training (CT) sessions over six months. The tasks included a range of activities from paper-and-pencil exercises to verbal learning exercises. The treated patients participated in in-group CT, while the control group did not. The results suggest that the CT program may improve the cognitive functions and potentially slow the cognitive decline in AD patients, at least temporarily.

Regarding MCI patients, studies conducted by Wenisch et al. [49], Belleville et al. [50], and Farini et al. [51] demonstrated that cognitive stimulation and rehabilitation programs significantly enhanced associative memory, verbal fluency, temporal orientation, episodic memory, and the overall cognitive and functional status.

Wenisch et al. [49] conducted a study to evaluate the effectiveness of cognitive stimulation programs in enhancing cognitive performance among individuals with MCI compared to cognitively normal older individuals. Both groups participated in cognitive exercises aimed at stimulating functions such as categorization, classification, and semantic association. The study found that the program improved associative memory, verbal fluency, and temporal orientation in MCI patients more effectively than in cognitively normal older individuals. Similarly, Belleville et al. [50] evaluated the effectiveness of cognitive training in improving episodic memory in individuals with MCI and adults with normal cognitive aging. The participants were divided into an intervention group and a waiting-list group. The intervention group, which included both MCI and cognitively normal participants, received cognitive training over eight weekly sessions. The study found significant improvements in delayed list recall, face–name association, subjective memory, and well-being among those who received the training in the intervention group. Farini et al. [51] conducted a study on the effects of an 8-week cognitive rehabilitation program using COGPACK (www.cogpack.com) [52] on individuals with mild cognitive impairment (MCI). The program included tasks such as verbal production and was applied to 10 subjects divided into two groups: amnestic MCI (a-MCI) and amnestic multi-domain MCI (amd-MCI). A control group, not undergoing the rehabilitation, was used for comparison. The study found that the cognitive rehabilitation program appeared to improve both cognitive function and functional status in participants with a-MCI and amd-MCI.

The NeuroPsychological Training (TNP) software [53,54] (including exercises of abstract reasoning, manipulation of concepts, relevant semantic and perceptive characteristics, and association abilities) has shown enhancing effects on the cognitive functioning of older individuals with AD and MCI.

Rozzini et al. [55] observed significant improvements in episodic memory and abstract reasoning, along with a reduction in behavioral disturbances, using TNP software [53,54] combined with cholinesterase inhibitors in MCI individuals. These improvements were particularly notable in cognitive areas like episodic memory and abstract reasoning, as well as in the reduction in depression, anxiety, and apathy. Cipriani et al. [56] conducted a study using the TNP cognitive training software [53,54] in patients with AD, MCI, and multiple system atrophy (MSA). The results demonstrated significant overall cognitive improvements in patients with AD and MCI. Specifically, AD patients showed enhancements in MMSE scores, verbal production, and executive functions, while MCI patients exhibited improved behavioral memory. However, no significant improvements were observed in the MSA group. Talassi et al. [57] conducted a study involving MCI and mild dementia (MD) patients to evaluate the efficacy of two types of non-pharmacological treatments: cognitive rehabilitation and physical rehabilitation. For the cognitive treatment, Talassi et al. [57] utilized the TNP software [53,54] alongside occupational therapy and behavioral training, obtaining significant improvements in visuospatial memory and physical performance and reductions in depression and anxiety symptoms in MCI patients.

Additionally, research comparing other rehabilitation programs for individuals with MCI and AD has demonstrated positive effects on cognitive functioning. Zaccarelli et al. [58] used the SOCIABLE platform (www.cognitivetraining.eu) [59,60,61] a computer-based cognitive training program featuring semantic task games such as “synonyms” and “antonyms,” and reported notable improvements in memory and executive functions. The study included cognitively intact older individuals, patients with MCI, and AD patients. The SOCIABLE intervention positively impacted global functioning, memory, and executive functions in individuals with mild AD and MCI, with patients also exhibiting enhancements in social and functional abilities. Moreover, several studies [62,63,64] utilized the GRADIOR cognitive rehabilitation program (INTRAS Foundation, Valladolid, Spain) [63], which includes exercises such as word comprehension and word recognition, in individuals with MCI and mild dementia. The patients showed improvements in various cognitive measures, including Cambridge Cognitive Examination (CAMCOG), Digit Span and Arithmetic from the Wechsler Adult Intelligence Scale (WAIS-III), Semantic Verbal Fluency, the Mini-Mental State Examination (MMSE), and the Trail Making Test (TMT), among others. They also exhibited high acceptance of the software and a positive attitude towards technology.

Most, if not all semantic tasks described here are standardized to a considerable extent, following a predefined sequence of steps (a script) for their implementation. Critically, the semantic references, such as stories and discussion topics, are often predefined as well. This can result in participants being unfamiliar with or uninterested in these topics, potentially reducing engagement and negatively affecting the outcomes. In contrast to this task-focused, standardized approach to semantic interventions, a person-centered approach allows the participants to choose their own topics, thereby encouraging expressive language in less-structured activities.

## 5. Person-Centered Approaches: Results from Linguistic–Semantic and Expressive Language Training

Expressive language refers to the productive aspect of linguistic communication. In the context of semantic training, individuals use expressive language whenever they share their thoughts, memories, opinions, or feelings with others through language, whether in written or in oral form. The so-called “communication-based programs”, as discussed below, particularly those aimed at AD patients, use expressive language as a tool to enhance both cognitive and psychosocial dimensions. Based on the available literature on the topic discussed below, one can conclude that compared to interventions centered around standardized language comprehension tasks, expressive language training has the following outcomes: (1) increases the possibility of addressing personally relevant semantic materials, thereby engaging the participants with meaningful topics [65,66]; (2) promotes social interaction through dyadic or group conversation [67,68].

Regarding (1), Bayles [65] suggests that engaging patients with personally relevant semantic materials during conversations, particularly older adults, including those with dementia, improves their ability to access their own knowledge as they repeatedly bring it to consciousness. Moreover, Peplau, in [66], further suggests that conversations may have a corrective effect on participants’ thought patterns by providing feedback, such as requests for clarification. In summary, using relevant semantic materials in expressive language contexts allows participants to choose their topics of interest, potentially leading to better cognitive outcomes.

Regarding (2), research has also emphasized the importance of interpersonal contexts, particularly in group settings. Sharing thoughts and experiences within a group can enhance the outcomes of cognitive training [67,68]. Hall et al. [67] examined the effects of Cognitive Stimulation Therapy (CST) in mild-to-moderate dementia patients on specific cognitive domains using a one-group pretest–posttest design. The study observed significant improvements in delayed verbal recall, visual memory, orientation, and auditory comprehension following the CST program. They suggested that CST may particularly benefit memory, comprehension of syntax, and orientation by enhancing neural pathways related to language processing. Notably, the absence of a control group limits the ability to draw definitive conclusions about the efficacy of CST. Spector et al.’s [68] study also explored the effects of CST on various aspects of cognition in people with dementia through a multi-center, single-blind, randomized controlled trial. The program incorporated, amongst other activities, themed sessions focusing on reminiscence. Control groups were included, where participants at each center continued their usual activities. The results showed that CST significantly improved the overall cognitive function, particularly language skills, while no significant changes were observed in memory, orientation, or praxis. These findings suggest that CST may be particularly effective in enhancing the language function, potentially leading to broader cognitive benefits.

Moreover, sharing thoughts and experiences within a group can enhance self-esteem and confidence [69], thereby influencing affective dimensions as well. Juarez’s study [69] aimed to assess a multicomponent cognitive stimulation therapy (SADEM) in patients with mild dementia. This controlled clinical trial included both an intervention group and a control group, with evaluations conducted longitudinally. The results showed significant improvements in cognitive outcomes and the Dementia Index post-intervention, with no observed disease progression by the study’s conclusion. These findings suggest that the therapy positively impacted cognitive and behavioral functions, as well as daily life activities, potentially delaying disease progression for up to two years.

The power of verbal expression to enhance affective and psychosocial dimensions has been further emphasized in studies of reminiscence therapy in healthy older adults [6]. This intervention modality combines conversation with personally relevant semantics, primarily focusing on the individual’s past [6,70]. It can vary in complexity, from simple recall and sharing of memories to more complex attempts to integrate past, present, and future experiences. Increased life satisfaction and quality of life have been reported as positive outcomes of this approach [6]. However, reminiscence therapy and expressive language approaches in the dementia literature are still largely missing. In the following sections, we will analyze the existing studies that have addressed these approaches.

### 5.1. Effects on Language Quality

In typical AD patients, a progressive decline in expressive language quality is common. These deficits often manifest as empty speech, reduced informational content, lexical retrieval difficulties accompanied by circumlocutions and paraphasias, the use of pronouns without clear antecedents, ideational perseveration, decreased coherence, frequent topic shifting, excessive verbosity, and poor comprehension of abstract language [71]. Early studies focused on the benefits of expressive language stimulation for language performance in AD patients, highlighting the advantages of structured over unstructured conversation.

For instance, Arkin and Mahendra [71] engaged an experimental group in activities such as picture description, associations with evocative words, proverb completion and interpretation, category fluency exercises, and advice and opinion questions, while the control group participated in unstructured conversation. The language outcome measures included a picture description task, a discourse battery, and proverb interpretation. The control group showed significant declines in the picture description tasks, while the experimental group maintained their baseline performance on these measures. Further, Mahendra and Arkin [72] used the same structured program and found that the four AD patients who completed the program over four years could maintain and even improve their discourse abilities. While no control groups were mentioned, the positive outcomes suggest the potential effectiveness of such interventions.

Similarly, Tappen et al. [70] implemented one-on-one structured conversation sessions centered on patients’ memories and analyzed the effects of adding a second layer of activity (walking and talking) to the primary activity (talking). They found smaller expressive language-related declines among patients who only engaged in conversation, suggesting that focusing entirely on self-expression may be key to preserving communication skills.

### 5.2. Effects on Cognitive, Mood, Emotional, and Functional Dimensions

Other studies have shown that fostering expressive language in AD patients may have broader impacts beyond language itself, extending to general cognition and affect. Bottino [73] conducted a study implementing a cognitive rehabilitation program for AD patients, comparing the cognitive effects of medication alone with those of medication combined with cognitive rehabilitation. The cognitive rehabilitation program, structured in group sessions, included activities such as discussing about one’s life, sharing past or recent experiences, discussing themes of common interest, or simulating daily life communication scenarios, such as having a conversation with a doctor. The group that participated in the cognitive rehabilitation sessions outperformed the control group in outcome measures, including MMSE scores and backward digit span scores (a measure of working memory).

Similarly, Olazarán et al. [74] implemented a cognitive program that combined socially and psychomotor-oriented activities. In addition to cognitive outcomes, the authors assessed the affective status of medicated AD patients before and after the intervention. The maintenance (as opposed to the decline) of cognitive function was more prevalent in the experimental group, where a higher number of participants also maintained or improved their affective status after one year of training.

Chapman et al. [75] examined the verbal, functional, and emotional impact of a program entirely based on conversational interaction. Again, a control group of medicated AD patients was compared with an experimental group that received both medication and cognitive-communication (conversational) training. The activities included participant-led discussions, interactive sessions about AD, and discussions centered on salient life stories. The experimental group exhibited less decline in verbal and functional abilities compared to the controls, as well as a decrease in negative emotional symptoms.

### 5.3. Focusing on Opinions, Not Facts

In contrast to the aforementioned cases of success, Onder et al. [76] found no impact of expressive language training within a group- and reality-based communication program for AD patients. The main feature of this approach is the emphasis on maintaining a connection with reality, including personal, temporal, and spatial orientation, as well as discussing news or topics of general interest. Although, to our knowledge, no direct comparisons are available between this program and other, less reality-focused programs, such as those based on reminiscence, a possible explanation for this lack of effectiveness may be that external facts do not appeal to AD patients.

One of the key principles of the Cognitive Stimulation Therapy (CST) approach, proposed by Spector et al. [77] and Spector et al. [78] in their program named “Making a Difference”, is the prioritization of opinions over facts. Other key principles include encouraging new ideas, thoughts, and associations, as well as stimulating language skills. The sessions include debates on topics of general interest, but primarily encourage the participants to share their opinions and personal experiences. They also incorporate structured cognitive challenges like word association, categorization, and number exercises, as well as physical activities. Alvares et al. [79] aimed to validate the CST for the Portuguese population in individuals with mild-to-moderate dementia. The research was a single-blind, multicenter, randomized controlled trial, with participants divided into an intervention group and a control group (which did not receive CST). The primary outcome measured was cognition, and the secondary outcomes included quality of life, communication, autonomy, anxiety, depression, and global functioning. The results indicated that the intervention group showed significant improvements in cognition, communication, behavior, and global dementia rating compared to the control group. However, there were no significant differences in quality of life, depression, or anxiety. The study concluded that CST is effective for the Portuguese population, particularly in enhancing cognitive function and related aspects in those with mild-to-moderate dementia.

The study by Capotosto et al. [80] investigated the effectiveness of the Italian version of Cognitive Stimulation Therapy (CST-IT) in improving cognitive functioning and quality of life for older adults with mild-to-moderate dementia. The participants were randomly assigned to either the CST-IT group or an active control group, which engaged in alternative activities. The CST-IT group showed better outcomes in maintaining cognitive function, particularly on the MMSE, and exhibited improvements in some cognitive, mood, and quality of life measures compared to the control group, which experienced cognitive decline.

The study by Carbone [81], using a similar design, also evaluated CST-IT in people with mild-to-moderate dementia. The participants were randomly assigned to either the CST-IT group or an active control group. The study assessed various domains, including cognitive functioning, language, mood, behavior, everyday life functioning, and quality of life, at the end of the treatment and three months later. They found benefits in mood, behavior, and quality of life measures, which were maintained at follow-up.

In summary, person-centered programs that value participants’ expressive language and personally relevant semantics have shown a positive impact not only on cognitive domains, including language, but also on social, affective, and functional areas.

## 6. Structured Expressive Language: The Question Club (CQ) Example

Expressive language offers benefits beyond cognition but can also present disadvantages due to a lack of structure, as, e.g., when working on language itself, structure tends to have a more positive impact (as noted above). Moreover, conversation requires a structured input for new learning to occur [70]. Furthermore, pure expression alone may not be sufficiently challenging for more ambitious cognitive goals, such as maintaining cognitive function in MCI or healthy older adults or even enhancing cognitive function in the latter. Some programs combine structured and unstructured tasks, but the question of whether these goals can be achieved within a single task that potentially integrates the strengths of both approaches remains (Table 1).

**Figure 1:**
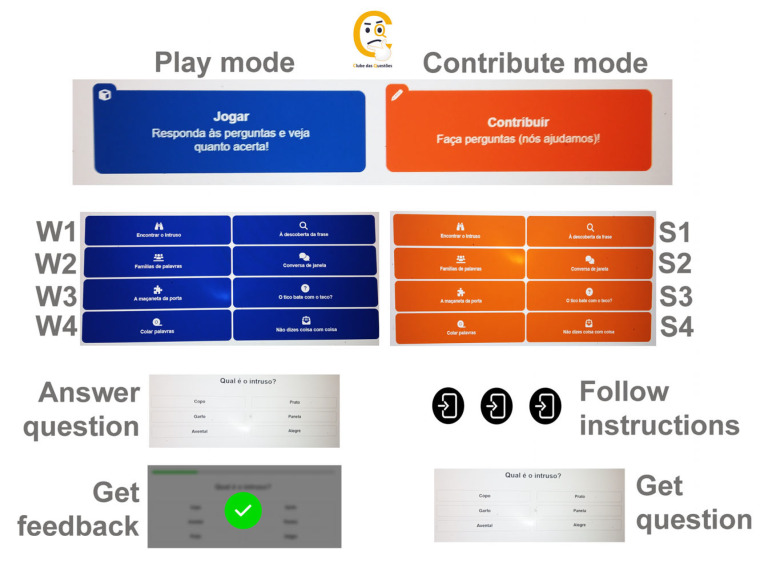
A schematic diagram of the CQ program. There are eight semantic tasks in each of the two modes (play and contribute): four focused on single-word meaning (W1–W4), and four requiring semantic integration at the sentence or text level (S1–S4). For a full description of the program, see Batista et al. [82].

We recently developed an online platform called the “question club” platform (www.clubedasquestoes.pt, assessed on 1 September 2024) (CQ) [82], where participants are invited to create their own multiple-choice quizzes using any of the eight activity formats included in the lexical–semantic stimulation program BOX (I Semantic Categories; II Syntagmatic and Paradigmatic Relationship; III Semantic Gradation; IV Adjectives and Exclamations; V Part-Whole Relationship; VI Anomalous Sentences; VII Semantic Definition; VIII Semantic Context) [35,69]. The participants may first try the activities as players, but the main focus is on creation. To facilitate this, the participants are guided through a thorough step-by-step procedure, always beginning by choosing a semantic referent—be it an object, a person, a living being important to them, a past experience, an opinion, etc. Several activity-dependent steps follow, leading the participants to complete the quiz they authored. The platform incorporates the participants’ input throughout the process, making the entire quiz dependent on it. For instance, in a categorization activity named “Find the intruder”, the participants are first asked to choose an object, a place, or a person important or familiar to them. If a participant chooses “dog”, the next question is “What kind of thing is a dog?”, to which they respond “animal” (category name). Then, follows a request to name other animals (other exemplars in the same category), and finally, they are asked to name something that is not an animal (the intruder). Therefore, the task of quiz creation begins by eliciting personally relevant semantics and follows a highly structured procedure continually fed by the participants’ input. Among the eight semantic tasks, four focused on single word meaning (W1-W4), while the other four required semantic integration at the sentence or text-level (S1-S4). We created new names for the activities (W1-Find the Intruder; W2-Word Families; W3-The Door’s Handle; W4-Glueing Words; S1-Discover the Sentence; S2-Chit-Chat; S3-Makes any Sense? S4-You Talk Foolishly!), which differ from those in the BOX program (for a full description of the tasks in the play and contribute mode, refer to Batista et al. [82]). For a brief overview of the program, see Figure 1.

Depending on the mode of implementation—single user, facilitator and participant, or facilitator and group—opportunities for social interaction and self-expression can easily emerge to varying degrees. Group administration allows the participants to share their experiences and knowledge with others when suggesting their personally relevant topics. The importance of this expressive component can be modulated according to the participants’ needs, and the components can be even distributed unequally among the group members: those with more severe impairments may be invited to share experiences or feelings, while those with better cognitive skills can work on that input to create the quiz. This approach favors the inclusion of various types of participants, extending the use of expressive language well beyond its most frequent application; for example, in dementia patients, going beyond self-expression, CQ aims to empower the participants by turning them into content creators and, ultimately, service providers.

## 7. Future Directions

The future directions in the field of semantic intervention are numerous and hold potential for yielding interesting, applicable, and beneficial results for both research and clinical practice. While targeting semantics appears to have a widespread positive impact, most programs—except perhaps for BOX—often include a variety of activities, both semantic and non-semantic, making it difficult to discern the specific impact of the semantic tasks. In such cases, it would be useful to validate the existing programs based on individual tasks (e.g., semantic vs. other tasks, and different types of semantic tasks) rather than evaluating entire packages. Although the idea of combining task-focused approaches with person-centered strategies in semantic interventions seems promising, there is little or no direct comparison between these methods. Research should prioritize direct comparisons between task-focused, person-centered, and hybrid approaches to semantics across different populations and objectives, particularly in the context of cognitive decline prevention and healthy aging. Furthermore, while expressive language interventions show great promise, their efficacy largely depends on the individual’s conversational skills. We need to optimize the benefits of expressive language, particularly by training pragmatic skills, to enable effective communication that ensures the speaker is both heard and understood, without exhausting the listener. Additionally, there is a need to develop corrective approaches that better structure communication and explore the most effective ways to share information.

## 8. Conclusions

In conclusion, literature on task-focused and person-centered semantic interventions for older adults, particularly in the context of dementia prevention and rehabilitation, is largely lacking. Existing studies on linguistic–semantic interventions have shown positive results across various outcome domains. However, further research is necessary to compare these two approaches and identify their respective strengths and potential synergies. For instance, combining task-focused and person-centered interventions, as exemplified by the CQ program, may offer a balanced approach that leverages the benefits of both methods.

## Figures and Tables

**Table 1:** Strengths of theTask-Focused and Person-Centered approaches.

Strengths of Task-Focused Approaches	Strengths of Person-Centered Approaches
Targeted Language Deficit Intervention	Personal Relevance and Engagement
Structured Learning	Social Interaction and Psychosocial Benefits
Transfer to General Knowledge and Comprehension Enhancement	Holistic Cognitive and Emotional Development

## Data Availability

No new data were created or analyzed in the preparation of this review.
